# Effect of mindfulness-based cognitive therapy vs. psychoeducational intervention on plasma brain-derived neurotrophic factor and cognitive function in bipolar patients: a randomized controlled trial

**DOI:** 10.3389/fpsyt.2023.1279342

**Published:** 2024-01-05

**Authors:** Diego Carracedo-Sanchidrian, Consuelo de Dios-Perrino, Carmen Bayon-Perez, Beatriz Rodriguez-Vega, Maria-Fe Bravo-Ortiz, Miguel Á. Ortega, Ana-Maria González-Pinto, Guillermo Lahera

**Affiliations:** ^1^Department of Psychiatry, University Hospital La Paz, Madrid, Spain; ^2^La Paz Hospital Institute for Health Research (IdiPAZ), Madrid, Spain; ^3^Autonomous University of Madrid (UAM), Madrid, Spain; ^4^Faculty of Medicine and Health Sciences, University of Alcalá, Madrid, Spain; ^5^CIBERSAM, Ramón y Cajal Institute of Sanitary Research (IRYCIS), Madrid, Spain; ^6^BIOARABA, UPV/EHU, CIBERSAM, Department of Psychiatry, Hospital Santiago Apostol, Vitoria, Spain; ^7^Principe de Asturias University Hospital, Alcalá, Spain

**Keywords:** bipolar disorder, mindfulness based cognitive therapy, psychoeducation, Brainderived neurotrophic factor, cognition, cognitive impairment

## Abstract

**Introduction:**

Few controlled trials have assessed the benefits of Mindfulness Based Cognitive Therapy (MBCT) on cognitive functions and brain-derived neurotrophic factor (BDNF) in bipolar disorder (BD). This study aims to evaluate the impact of MBCT adjunctive treatment on these variables. Main hypothesis was that MBCT would improve cognitive functioning and BDNF more than Psychoeducation and TAU.

**Methods:**

Randomized, multicenter, prospective and single-blinded trial. Included BD outpatients randomly assigned to three treatment arms: MBCT plus treatment as usual (TAU), Psychoeducation plus Tau and TAU. Cognitive functions were assessed with Continuous Performance Test-III, Stroop Test, Trail Making Test, Digit Span and Letter-Number Sequencing from Wechsler Adult Intelligence Scale III, Face Emotion Identification Task and Face Emotion Discrimination Task. BDNF serum level was measured with ELISA. Patients were assessed at baseline, 8 weeks and 6 months.

**Results:**

Eighty-four patients were recruited (TAU = 10, Psychoeducation = 34, MBCT = 40). No significant differences between treatment groups were found. MBCT does not achieve better results than Psychoeducation or TAU.

**Discussion:**

Being Psychoeducation and TAU efficient interventions, as well as the scarce duration of a more complex intervention, such as MBCT, are suggested as explanatory variables of these results.

**Trial registration:**

ClinicalTrials.gov: NCT02133170. Registered 04/30/2014.

## 1 Introduction

Bipolar Disorder (BD) has a prevalence of 0.5–5% (1), and is one of the mental disorders that generates significant interference and disability (2), including a high risk of suicide (3). The functional impairment of this disorder appears to be related to the associated cognitive deficits (4, 5), which are diverse (6–8) and manifest not only during affective episodes but also during remission (9). Although it is not clear whether the impairment is progressive (10), deficits have been found in domains such as attention, executive functions (11), verbal memory, psychomotor speed (12) and social cognition (13–15). In fact, despite clinical improvement being able to alleviate cognitive difficulties, a neuropsychological assessment is recommended as a routine clinical practice (16).

Various interventions have been investigated that go beyond symptom reduction and aim to improve functioning, quality of life, and cognitive deficits (17). Some of these interventions are preventive and health-focused, such as medication adherence, physical exercise, comorbidity control, or cognitive stimulation (5). However, the bulk of the research has sought specific interventions. In a recent review of controlled studies analyzing 16 pro-cognitive interventions, it was concluded that none of them have robustly and independently demonstrated cognitive benefits in adults with BD (18). In this line of inquiry, the working group led by Torrent (19) conducted a controlled and randomized study comparing Cognitive Remediation (CR) with psychoeducation and standard treatment in a total of 183 patients. The study found that group-based cognitive remediation applied over 21 weeks was superior to standard treatment in terms of functional improvement, although not in comparison to psychoeducation. Demant and colleagues also applied CR for 12 weeks to 23 patients with BD, comparing them with standard treatment, but in this case, no differences were found (20). Similarly, using CR, but in this case through 70 h of computerized treatment, Lewandosky and colleagues (21) did find improvement in various cognitive domains such as memory, visual memory, and processing speed in a controlled study comparing treatment in 39 patients with 33 controls. CR was also employed by the team led by Veeh (22) in a naturalistic study where 26 patients with BD participated in a computerized program for 12 weeks. In comparison to the control group, there appeared to be improvement in executive functions following the treatment, although the sample size was very limited. Lastly, a variation of CR, known as Action-Based Cognitive Remediation (ABCR), was tested in remitted patients with BD, but it only achieved short-term improvement in executive functions and not in subsequent follow-ups when compared to controls in a study involving a total of 61 patients (23).

Other cognitive interventions have been used as well. Gomez et al. (24) conducted a controlled study with 39 patients, comparing Cognitive-Behavioral Rehabilitation with treatment as usual (TAU). After 12 weeks of treatment, they observed improvement in the treatment group in reaction time, visual memory, and emotion recognition, although it should be noted that this study also had a small sample size. On the other hand, Lahera et al. (13) applied an Interaction and Social Cognition Training to 37 patients with BD and Schizoaffective Disorder, finding improvement in social cognition.

The mentioned interventions focus on the rehabilitation of cognitive functions, but there are others that aim to combine clinical and cognitive benefits, such as Mindfulness-Based Cognitive Therapy (MBCT). A recent meta-analysis reviewing 10 controlled studies concludes that MBCT appears to be effective in reducing symptoms of anxiety and depression in this population, although the evidence is still inconsistent and further research is needed (25). Nevertheless, this intervention seems to have demonstrated viability and no negative effects for this population (26), and some of its benefits persist years later (27). MBCT has shown some benefits in cognitive functions such as working memory, autobiographical memory, and cognitive flexibility in the general population. However, the benefit in attention and other executive functions has not yet been demonstrated (28). There are few studies on the benefits of MBCT among the population with BD, but there are some, such as the study conducted by Stange (29). In this study, after 12 sessions of MBCT, patients reported improvement in executive functions, memory, and the ability to initiate and complete tasks. It should be noted that this was a small pilot study with 9 patients and no control group, but with a follow-up assessment conducted 3 months after treatment. Subsequently, in a controlled intervention using MRI, it was found that after 8 sessions of MBCT, there was an increase in activation in the prefrontal cortex, an area associated with executive functions and cognitive flexibility. However, it is important to note that this study compared the results with only 9 patients in the control group (30). There are more recent studies that, although not using the MBCT protocol specifically, have employed mindfulness-based interventions with other components such as psychoeducation and cognitive strategies, and have found improvements in reported cognitive functioning (31).

One of the variables that has been associated with cognitive impairment in BD is the Brain-Derived Neurotrophic Factor (BDNF) (32, 33). BDNF is a neurotrophin that plays an important role in the survival, growth, and maintenance of neurons in key brain circuits related to emotional and cognitive function (34, 35). BDN factor is notably abundant in the hippocampus and the brain cortex, both of which are closely associated with the regulation of mood and cognition. As BDNF is released form platelets during blood coagulation, its levels are readily detectable in human serum. Consequently, peripheral BDNF levels have the potential to serve as an indicator of brain BDNF levels, which tend to be lower in individuals with BD compared to those in good health (36). However, its relationship with cognitive impairment in bipolar disorder is not yet fully understood (37). BDNF has also been associated with affective episodes (38) and has been considered a potential differential marker between individuals with bipolar disorder and healthy controls (39). However, a recent review and meta-analysis of 35 studies highlights the limitations of the available evidence, suggesting moderate conclusions regarding the role of peripheral BDNF as a biomarker in bipolar disorder and calls for further research in this area (40).

Although limited, research has been conducted on whether Mindfulness-Based Interventions (MBIs) can affect plasma levels of BDNF. A literature review of 15 studies (41) and a systematic review and meta-analysis of 11 controlled studies (42), came to the same conclusion that, although the evidence is limited due to the heterogeneity and small size of the studies, the data suggest that MBIs may increase peripheral BDNF levels. Research on the population with bipolar disorder is even scarcer. In an initial study, Wiener’s team (43) assessed the impact of adding psychoeducation to the standard pharmacological treatment on BDNF, Nerve Growth Factor (NGF), and Glial Cell Line-Derived Neurotrophic Factor (GDNF) in a controlled study involving 39 young patients. The findings revealed changes in GDNF but not in the other two trophic factors. In a second study in this line (44), this time utilizing Functional Remediation, no differences were found between this intervention, psychoeducation, and standard treatment in BDNF levels among euthymic patients with bipolar disorder. However, improvements were observed in psychosocial functioning. Using an adapted version of the MBCT protocol, Augmented Mindfulness-Based Cognitive Therapy (45), an increase in BDNF and NGF was found in the experimental group in a controlled study involving 160 patients with unipolar depression.

The present study aims to analyze the impact of MBCT on cognitive abilities, specifically attention, working memory, and social cognition, as well as BDNF levels in individuals with BD.

## 2 Materials and methods

### 2.1 Design

The study was a randomized controlled trial (NCT02133170), prospective, multicenter, and single-blind, involving patients with BD and subclinical depressive symptoms. The inclusion and exclusion criteria, type of intervention, and detailed methodology have been previously published (46). In summary, patients with BD and subclinical depressive symptoms were recruited from mental health centers, hospitals, private clinics, and associations through advertisements and referrals from their psychiatrists. The inclusion criteria required participants to be between 18 and 65 years old, diagnosed with BD, receiving stable pharmacological treatment according to clinical practice guidelines, and scoring between ≥8 and ≤ 19 on the Hamilton Depression Rating Scale and < 8 on the Young Mania Rating Scale. Participants were excluded from the study if they had experienced an acute episode within 12 weeks prior to the study, had a risk of suicide, had received psychotherapy or psychoeducation in the past 5 years, had intellectual disability, were pregnant, or had participated in another study within that could involve psychotherapy or drugs the last 4 weeks.

**Figure 1:**
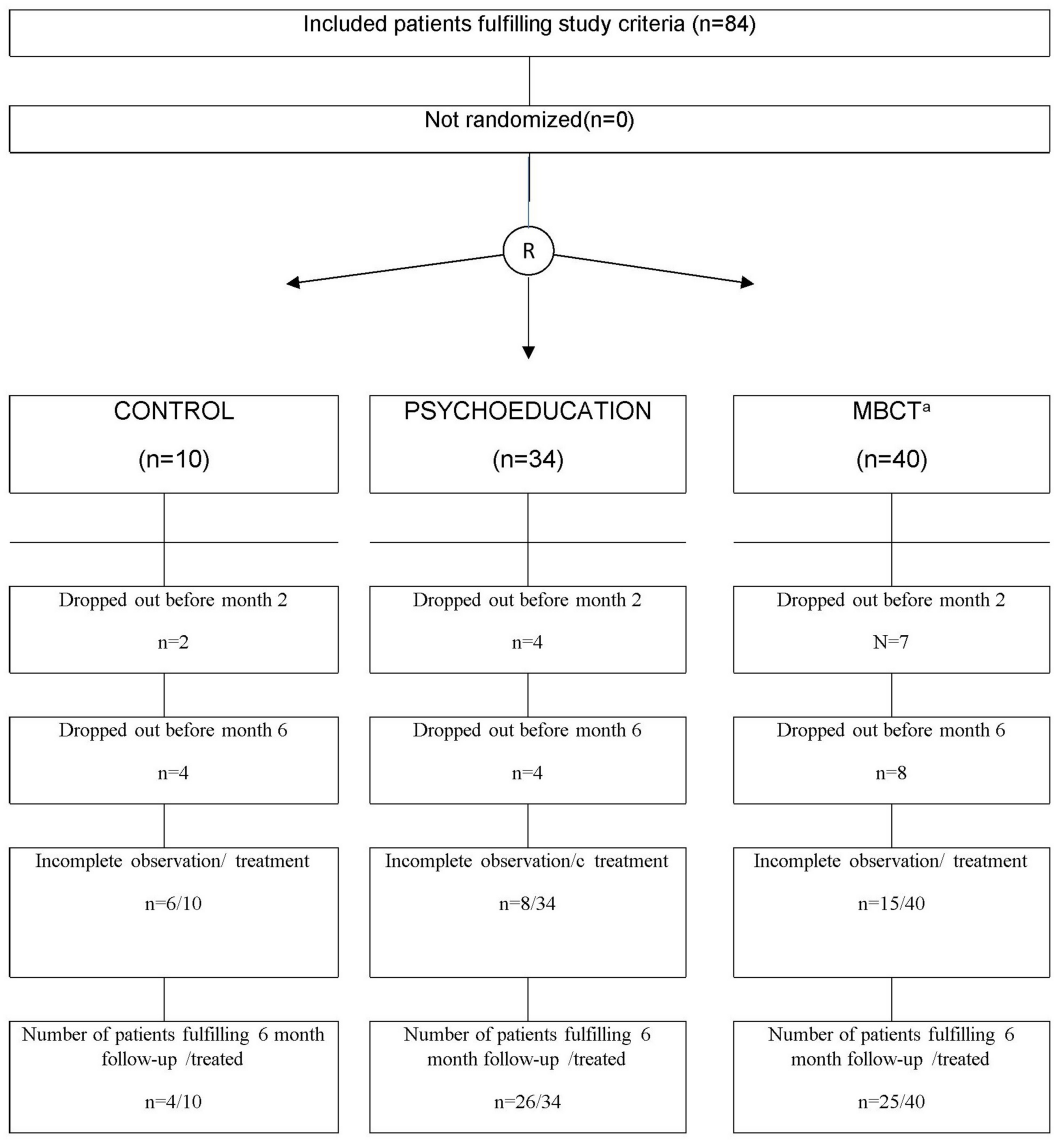
Patient’s disposal. ^a^Mindfulness-based cognitive therapy.

After screening 136 patients, 84 were included in the study, and 55 completed the 6-month follow-up. Among these, 46 participants were recruited from La Paz University Hospital area, and 38 from Santiago Apóstol Hospital area. A semi-structured interview based on the Mini International Neuropsychiatric Interview Plus revised was applied to select and assess patients. Sociodemographic and clinical data included gender, age, marital status, educational status and occupation, age at onset of BD, age at first hospitalization, history of psychotic symptoms, polarity of the first episode, total number and type of previous episodes, number of hospitalizations, course specifiers according to DSM-5, bipolar subtype, physical comorbidities, family psychiatric history, family history of affective disorder, family history of completed suicide, history of suicidal ideation, number of suicide attempts, and history of drug misuse. Evaluators were psychiatrists and clinical psychologists blind to treatment. They underwent training in the utilization of assessment scales to mitigate any potential inter-rater variability. Random Allocation Software was used to randomize the participants into three groups: Usual pharmacological treatment as indicated by clinical guidelines (47) (TAU), psychoeducation plus TAU, and Mindfulness-Based Cognitive Therapy (MBCT) plus TAU, in a ratio of 1:4:4. Accordingly, 10 patients received TAU, 34 received psychoeducation plus TAU, and 40 received MBCT plus TAU (Figure 1). All participants were assessed at baseline (V1), at the end of the treatment (V2), and at the 6-month follow-up (V3). For more detailed information, refer to (48).

The study followed the CONSORT checklist and the international guidelines of the Declaration of Helsinki. It has obtained approval from the Ethics Committee of La Paz University Hospital (Madrid) and the autonomous community of the Basque Country. Participant privacy has been ensured in accordance with the LOPD 3/2018 law.

### 2.2 Intervention

The psychoeducation intervention was delivered in a group format, with 2-h sessions held weekly for 8 weeks. The sessions covered topics such as prodromal detection, relapse prevention, understanding the illness, and treatment adherence, following a standardized manual (49). The MBCT intervention is a manualized training program that integrates aspects of Cognitive Behavioral Therapy and meditation components of mindfulness-based stress reduction program. It consisted of 8 weekly sessions, each lasting 90 min, following the original protocol (50, 51), which combines elements of mindfulness-based interventions with cognitive-behavioral therapy. The sessions were conducted by expert therapists and were video-recorded to ensure fidelity using the Mindfulness-Based Cognitive Therapy Adherence Scale (MBCT-AS). Brief written information about BD was given to the patients at the beginning of the therapy.

### 2.3 Materials

Sustained attention was assessed using the Continuous Performance Test-III (CPT-III). CPT-III is a 14 min computerized test during which the patient must maintain attention to the task. This task involves pressing the space bar on the keyboard when a letter appears (referred to as the “target”), except when the letter X is displayed (referred to as the “non-target”). Stimulus appears on the screen with varying time frequency and the patient must respond until the end of the task. A training phase is incorporated to ensure that the patient comprehends the task. CPT3 allows to measure correct answers, omissions (no response to target), commissions (response to non-target), effect of fatigue and vigilance level. CPT reliability in test–retest, according to its manual is 0.87 (52).

Selective attention was evaluated using the Stroop Color Word test. The Stroop Test is a brief three-page assessment that can be administered in just a few minutes. The first task involves reading the words “red,” “green,” and “blue” printed in black ink. The second task requires identifying the ink color (red, green, or blue) of a “XXXX” item. The third and interference task entails reading the words “red,” “green,” and “blue” presented in red, green, and blue ink, with the challenge that the word and the ink colors do not correspond. Each task is completed in 45 s. The reliability of the test is very consistent, according to the manual is 0.85, 0.81 and 0.69, for each version (53).

The Trail Making Test consists of two parts: Part A, in which the goal is to connect, through lines and consecutively, 25 randomly distributed numbers on a sheet. In Part B, the connection has to follow the same logic but alternating between numbers and letters. Pre-trials are conducted in both parts of the test. TMT was also employed to assess the ability to selectively attend and shift focus, and has shown good levels of reliability (54).

Working memory was assessed using the digit and letter-number sequencing subtests of the Wechsler Adult Intelligence Scale-III (WAIS-III) (55). The WAIS-III is composed by various tests. For this cognitive assessment the Digit and Number sequencing subtest was used as a working memory measure. In this test the patient listens to increasing sequences of letters and numbers and is required to repeat them in a sequential and organized manner. The more digits and letters in order the patient repeats, the more working memory capacity is assumed. WAIS-III has demonstrated a good internal consistency reliability when used to evaluate cognitive functions in clinical groups (56).

Social cognition was assessed using the Face Emotion Identification Task (FEIT) and the Face Emotion Discrimination Task (FEDT) (57). The FEIT involves evaluating emotions in images, consists of 19 photographs of individuals, faces depicting one emotion of a total of six (sadness, anger, surprise, happiness, fear or shame). It could be presented on a computer, as in our case. The patient must choose the correct emotion in each picture. Regarding FEDT, it involves 30 pairs of images with two people depicting one of six emotions (same as in FEIT). In this task, pairs of individuals are simultaneously presented, and the objective is to determine whether they are displaying the same or different emotions. As FEIT, it was presented on a computer screen. Both FEDT and FEIT have demonstrated good reliability in clinical population (58).

Patients were informed that the study involved measurement of Serum levels of Brain-Derived Neurotrophic Factor (BDNF) and its extraction method. If they refused, they were completely excluded from the study. The blood draw was always performed at the usual time for these procedures in the hospitals, between 8 and 9 a.m. The sample was centrifuged to separate the serum and then stored in the refrigerators of each of the hospitals until analyzed by ELISA, following the instructions provided by the manufacturer (59). The procedure was repeated using the same method on all three occasions for all patients.

### 2.4 Statistical analysis

Qualitative variables among intervention groups or among other breakdown factors were compared using Fisher or Χ^2^ tests. For quantitative variables ANOVA, Student t test, Kruskal–Wallis or Mann–Whitney, depending on the results of the Kolmogorov–Smirnov–Lilliefords normality tests and the number of categories in the comparisons. Intention-to-treat analysis was done with last observation carried forward imputation method for missing data substitution. Change of variables during follow-up was performed with a General Linear Model, ANOVA with repeated measures with two factors and repeated measures in one factor (Split Plot), with Bonferroni corrections for the control of the multiple comparisons error, which also allowed assessing if statistically significant changes where present according to intervention group. Data were analyzed using SPSS 25.0 and two-tailed *p* < 0.05 considered significant.

The initial sample size was expected to be of 140 patients, allowing a power of 80%. The *post-hoc* power analysis for three levels in the treatment factor group, and two levels of follow-up from baseline to 6 months, of fixed effects analysis of variance for a total of 84 cases, yields a power of 52% to reach significance with an effect size (*f* = 0.25) between treatment groups, and 63% to reach significance in the total group follow-up (*f* = 0.25), and a power of 52% for the interaction between factors (Sample Power, IBM-SPSS).

## 3 Results

### 3.1 Sample characteristics

There were no statistically significant differences between the groups in social variables or in most clinical variables. Differences were found only for the melancholic specifier for the last episode (*p* = 0.04) and in family history of affective disorders (*p* = 0.04). The remaining detailed sociodemographic data is show in Table 1.

**Table 1:** Sociodemographic and clinical characteristics.

		Treatment group							
		Control		Psycho-education		MBCT^a^		Total	
		*n*	%	*n*	%	*n*	%	*n*	%
Marital status	Single	5	50.0%	14	41.2%	20	50.0%	39	46.4%
	Married	2	20.0%	11	32.4%	12	30.0%	25	29.8%
	Separated-divorced	3	30.0%	9	26.5%	8	20.0%	20	23.8%
	Widow	0	0.0%	0	0.0%	0	0.0%	0	0.0%
Job status	Working	0	0.0%	13	38.2%	14	35.0%	27	32.1%
	Unemployed	6	60.0%	6	17.6%	13	32.5%	25	29.8%
	Disability	4	40.0%	13	38.2%	12	30.0%	29	34.5%
	Retired	0	0.0%	1	2.9%	1	2.5%	2	2.4%
	Student	0	0.0%	1	2.9%	0	0.0%	1	1.2%
Drug use	Tobacco	6	60.0%	16	47.1%	18	45.0%	40	47.6&
	Caffeine	5	50.0%	17	50.0%	25	62.5%	47	56.0%
	Alcohol (occasionally)	7	70.0%	13	39.4%	20	50.0%	40	48.2%
	Past alcohol use disorder	0	0.0%	2	6.1%	1	2.5%	3	3.6%
	Past cannabis use disorder	5	50.0%	4	11.8%	7	17.5%	16	19.0%
	Past cocaine use disorder	1	10.0%	1	2.9%	5	12.5%	7	8.3%
	Past hallucinogen use disorder	0	0.0%	2	5.9%	4	10.0%	6	7.1%
	Other drug abuse	0	0.0%	0	0.0%	4	10.5%	4	5.1%
DSM-5 diagnosis	BD I	7	70.0%	25	73.5%	29	72.5%	61	72.6%
	BD II	3	30.0%	9	26.5%	10	25.0%	22	26.2%
	BD not otherwise specified	0	0.0%	0	0.0%	1	2.5%	1	1.2%
Course specifiers (DSM-5)	Anxious distress*	6	60.0%	22	73.3%	27	71.1%	55	70.5%
	Seasonal pattern (life)	3	30.0%	2	6.7%	8	21.1%	13	16.7%
	Rapid cycling (life)	2	20.0%	3	10.0%	11	28.2%	16	20.3%
	With catatonia*	0	0.0%	0	0.0%	1	2.6%	1	1.3%
	Melancholic Features*†	3	30.0%	6	20.0%	18	48.6%	27	35.1%
	Atypical features*	1	10.0%	3	10.3%	3	7.9%	7	9.1%
	Mixed features*	1	10.0%	5	16.7%	5	13.2%	11	14.1%
	Psychotic symptoms*	1	10.0%	8	26.7%	7	18.4%	16	20.5%
	Postpartum-onset*	0	0.0%	0	0.0%	2	5.3%	2	2.6%
Psychosis⸆	Yes	3	33.3%	6	33.3%	10	66.7%	19	45.2%
Initial polarity	Hypo/Mania	4	40.0%	14	43.8%	9	23%	27	33.4%
	Depression	6	60.0%	18	56.3%	29	74.4%	53	65.4%
	Mixed episode	0	0.0%	0	0.0%	1	2.6%	1	1.2%
Suicide ideas⸆	Yes	6	60.0%	18	52.9%	25	62.5%	49	58.3%
Suicide attempt⸆	Yes	5	50.0%	8	23.5%	14	35.0%	27	32.1%
Family psychiatric⸆	Yes	9	90.0%	22	64.7%	26	65.%	57	67.9%
Family affective disorder†	Yes	9	90.0%	16	47.1%	19	47.5%	44	52.4%
Family history of completed suicide	Yes	4	40.0%	4	11.8%	6	15.0%	14	16.7%
Years from diagnosis		10	14.9	34	13.08	40	14.03	84	13.75
N° affective episodes		10	11.8	34	8.4	40	12.1	84	10.5

*Last episode; ⸆Previous history † *p* = 0.04. ^a^mindfulness-based cognitive therapy.

### 3.2 Cognitive outcomes

#### 3.2.1 Attention

- CPT-III: There were no significant differences in omissions or baseline; nor between the three visits in the total sample or between groups at any of the three visits (Supplementary Tables S1, S2). Significant differences were observed only in commission errors between V1 and V3 in the total group (*p* = 0.04), but not in the baseline or between groups. Regarding reaction time (correct responses), there were no differences in the baseline, nor in the three visits in the overall sample or between groups (Supplementary Tables S3, S4).
- Stroop: Significant differences were found in color reading speed between V1 and V3 (*p* = 0.025) and between V2 and V3 (*p* = 0.000) in the total group. Furthermore, significant differences were also observed between the control group and the psychoeducation group (*p* = 0.01), as well as between the control group and the MBCT group at V3 (*p* = 0.01; Table 2). No significant differences were found in word reading speed, neither in the baseline nor between visits or treatment groups (Table 2). Regarding interference, no differences were found between visits in the total group or between groups (Supplementary Tables S5, S6).
- TMT: Significant differences were found in the execution time of the TMT-A between the control group and the psychoeducation group at V1 (*p* = 0.03). Additionally, there were significant differences in the number of errors committed at V3 between groups (*p* = 0.00 for control vs. psychoeducation, *p* = 0.00 for control vs. MBCT; Table 2). No significant differences were observed in the rest of the comparisons (Table 2).

**Table 2:** Significant outcomes in attention.

		Outcomes											
		CPT-III commissions		Stroop Color		Stroop word reading		TMT-A time		TMT-A mistakes		TMT-B time	
		Mean difference	*p*	Mean difference	*p*	Mean difference	*p*	Mean difference	*p*	Mean difference	*p*	Mean difference	*p*
V	Treatment group												
V1-V2	Whole sample	0.33	1.00	−3.02	0.43	−3.97	0.20	1.34	1.00	0.00	1.00	15.17	0.04
V2-V3		4.05	0.08	10.01	0.00	5.66	0.01	3.59	0.16	0.08	1.00	4.11	1.00
V1-V3		4.43	0.04	6.98	0.02	1.68	1.00	1.93	0.23	0.08	1.00	19.28	0.02
V1	Control vs. psychoeducation	6.22	0.64	7.02	1.00	7.95	0.90	−21.10	0.03	0.84	0.42	−2.98	1.00
	Control vs. MBCT^a^	6.65	0.56	4.53	1.00	4.98	1.00	−14.13	0.26	0.85	0.41	−10.96	1.00
	Psychoeducation vs. MBCT	0.42	1.00	−2.49	1.00	−2.96	1.00	6.97	0.37	0.00	1.00	−7.98	1.00
V2	Control vs. psychoeducation	2.06	1.00	9.21	0.53	11.78	0.25	−19.25	0.13	0.94	0.19	−30.37	0.42
	Control vs. MBCT	7.31	0.74	9.26	0.53	11.21	0.29	−14.30	0.39	0.75	0.41	−27.92	0.52
	Psychoeducation vs. MBCT	5.25	0.42	0.04	1.00	−0.56	1.00	4.94	1.00	−0.19	0.35	2.45	1.00
V3	Control vs. psychoeducation	0.57	1.00	−24.28	0.01	−8.53	0.94	−15.14	0.24	0.94	0.00	−20.93	0.86
	Control vs. MBCT	1.92	1.00	−24.00	0.01	−8.86	089	−9.03	0.87	1.00	0.00	−21.41	0.82
	Psychoeducation vs. MBCT	1.34	1.00	−0.28	1.00	−0.33	100	6.11	0.58	0.05	0.93	−0.47	1.00

^a^Mindfulness-based cognitive therapy.

Significant differences were found in the execution time of the TMT-B between V1 and V2 (*p* = 0.04) and between V1 and V3 (*p* = 0.02; Table 2), but not between treatment groups. Regarding errors, no significant differences were observed (Supplementary Tables S7, S8).

#### 3.2.2 Working memory and executive functions

- Digits: No significant differences were found between visits or between treatment groups (Supplementary Tables S9, S10).
- Letters and Numbers: There were no differences in this measure between visits in the total group or between treatment groups (Supplementary Tables S11, S12).

#### 3.2.3 Social cognition

- FEIT: There were no significant differences in the total group between visits, nor between treatment groups (Supplementary Tables S13, S14).
- FEDT: There were no differences in the total group between visits in this measure. However, there were differences between groups in the post-treatment measure (V2) between MBCT and psychoeducation (*p* = 0.02) although no differences were observed in any other comparisons (Table 3).

**Table 3:** Significant outcomes in social cognition.

Outcomes			
FEDT			
		Mean difference	*p*
V	Treatment group		
V1-V2	Whole sample	−0.91	0.23
V2-V3		−0.11	1.00
V1-V3		−1.03	0.21
V1	Control vs. psychoeducation	0.23	1.00
	Control vs. MBCT^a^	0.77	1.00
	Psychoeducation vs. MBCT	0.53	1.00
V2	Control vs. psychoeducation	0.98	1.00
	Control vs. MBCT	2.52	0.53
	Psychoeducation vs. MBCT	1.53	0.02
V3	Control vs. psychoeducation	1.64	0.58
	Control vs. MBCT	1.52	0.68
	Psychoeducation vs. MBCT	−0.11	1.00

^a^Mindfulness-based cognitive therapy.

### 3.3 BDNF outcomes

- No statistically significant differences were observed either between study periods or between treatment groups within each period (Supplementary Tables S15, S16).

## 4 Discussion

The aim of this study was to assess the impact of adding MBCT to TAU compared to Psychoeducation and TAU, and TAU alone, on cognitive functions and BDNF in individuals with BD. The findings of this randomized clinical trial indicate that after 8 weeks of treatment with MBCT, only improvements in attention are achieved, although these improvements appear to be attainable through Psychoeducation as well, albeit to a lesser extent, with TAU. There are no improvements in executive functions or working memory, and although an initial improvement in social cognition is observed with MBCT compared to Psychoeducation, the difference fades away at the 6-month follow-up. No changes have been found in plasma levels of BDNF in any of the treatment groups.

These predominantly negative results can be explained by various reasons. Firstly, all the interventions studied may produce some degree of clinical improvement (17), which could reduce the interference of symptoms on attentional processes. The TAU consisted of a high-quality approach, recommended by international clinical practice guidelines, and Psychoeducation may indirectly impact cognitive functions (60). This is consistent with some studies where similar results are found among different interventions, including those specifically targeted at cognitive rehabilitation (CR) (18, 19). It is also reasonable to consider that the subjective improvement in perceived cognitive functions found in some studies (30) may be attributed to the same phenomenon. In this regard, the fact that our sample presented subclinical depressive symptoms could have also interfered with the potential cognitive improvement.

Regarding the studies that did find improvements in cognitive function, the majority used CR or an adaptation of it, but these studies had small sample sizes (24), or were applied to patients with pre-existing cognitive impairment (22), or the improvements declined during follow-up (22). In the case of Lewandosky (20), perhaps due to the population being individuals with BD with psychosis, they obtained more benefit from the CR intervention, as its effectiveness has been established in populations with psychosis (61). It is worth noting that Stange (28) did find improvements with MBCT in a sample with BD, but in a study with only 8 patients, without a control group, and with a follow-up of only 3 months, making it difficult to generalize these results. It is possible that the longer duration of their intervention (12 sessions instead of 8) influenced these findings.

Neither MBCT nor Psychoeducation were more effective than TAU in improving social cognition in patients with BD. The intervention conducted by Lahera (13) specifically targeted social cognition, suggesting that a broad-spectrum intervention like MBCT or Psychoeducation may not yield such specific benefits, but they could be achieved through more targeted interventions. Additionally, measuring social cognition solely with the FEIT and FEDT tests may have been insufficient due to their low ecological validity.

Lastly, regarding BDNF, our results are consistent with previous studies where no changes were found, whether with Psychoeducation (43), or CR (44) applied to individuals with BD. In this regard, with an adapted version of MBCT for unipolar depression, an increase in plasma BDNF has been found (45), which is consistent with the fact that MBCT has demonstrated clear evidence for clinical symptoms in unipolar depression (62).

In summary, MBCT-based interventions are feasible in this population, although an 8-week application yields limited cognitive improvements that fade over time. This should prompt clinicians and researchers to strive for more specific or extended interventions that can provide greater benefits while aiming to maintain the efficiency of group intervention.

To the best of our knowledge, the present study is one of the largest controlled and randomized trials evaluating the efficacy of MBCT on cognitive functions and BDNF levels in individuals with BD, with two active treatment groups using structured protocols and a 6-month follow-up. Although the intended ratio of 1:2:2 could not be maintained and was instead 1:4:4, this does not affect the results (48), and this distribution is sometimes even considered advantageous by some authors (63).

Regarding the limitations of the study, the fact that both TAU and Psychoeducation are effective interventions for BD makes it difficult for MBCT to surpass them, just as other interventions like CR have also failed to do so. Additionally, subclinical depressive symptoms may have been a variable that limited the utilization of the intervention, as MBCT requires more proactivity and work outside of sessions compared to other interventions. Lastly, the lack of parallel assessment measures prevents ruling out the possibility that some of the improvements detected in the overall group may be due to a learning effect, as the patients underwent the same evaluation three times.

Future research should aim to investigate whether an increase in the number of sessions leads to stable cognitive improvements and changes in BDNF values, as suggested by some data. Furthermore, exploring whether more specific interventions are more beneficial in specific areas would be valuable. It is also important to employ evaluation measures with parallel forms to eliminate the potential learning effect.

## Data availability statement

The original contributions presented in the study are included in the article/Supplementary material, further inquiries can be directed to the corresponding author.

## Ethics statement

The studies involving humans were approved by Ethics Committee of La Paz University Hospital (Madrid) and the autonomous community of the Basque Country. The studies were conducted in accordance with the local legislation and institutional requirements. The participants provided their written informed consent to participate in this study.

## Author contributions

DC-S: Writing – original draft, Writing – review & editing. CD-P: Conceptualization, Funding acquisition, Investigation, Methodology, Project administration, Supervision, Writing – review & editing. CB-P: Investigation, Supervision, Writing – review & editing. BR-V: Conceptualization, Investigation, Supervision, Writing – review & editing. M-FB-O: Resources, Supervision, Writing – review & editing. MO: Funding acquisition, Writing – review & editing. A-MG-P: Conceptualization, Writing – review & editing. GL: Conceptualization, Funding acquisition, Investigation, Methodology, Supervision, Writing – original draft, Writing – review & editing.
