# Supplementary material for: Effect of mindfulness-based cognitive therapy vs. psychoeducational intervention on plasma brain-derived neurotrophic factor and cognitive function in bipolar patients: a randomized controlled trial
Source: Front Psychiatry. 2024 Jan 5;14:1279342. doi: 10.3389/fpsyt.2023.1279342 (PMC10796620; doi:10.3389/fpsyt.2023.1279342)
Supplement: Supplementary file 1 [file Table_1.DOC]

Supplementary data and tables

**CPT-III omissions**

| **Pairwise Comparisons** | | | | | | |
| --- | --- | --- | --- | --- | --- | --- |
| (I) Visit | (J) Visit | Mean difference  (I-J) | Dev. Error | Sig^a^ | 95% confidence interval for difference^a^ | |
|  |  |  |  |  | Lower limit | Upper limit |
| 1 | 2 | 14.04 | 19.86 | 1.00 | -35.30 | 63.40 |
|  | 3 | 17.34 | 19.93 | 1.00 | -32.18 | 66.88 |
| 2 | 1 | -14.04 | 19.86 | 1.00 | -63.40 | 35.30 |
|  | 3 | 3.30 | 2.76 | 0.71 | -3.55 | 10.16 |
| 3 | 1 | -17.34 | 19.93 | 1.00 | -66.88 | 32.18 |
|  | 2 | -3.30 | 2.76 | 0.71 | -10.16 | 3.55 |
| Based on estimated marginal means. | | | | | | |
| a, Adjustment for multiple comparisons: Bonferroni.  Table 1s. CPT-III omissions, total group. | | | | | | |

| **Pairwise Comparisons** | | | | | | | |
| --- | --- | --- | --- | --- | --- | --- | --- |
| Visit | (I) Treatment group | (J) Treatment group | Mean difference (I-J) | Dev. Error | Sig^a^ | 95% confidence interval for difference^a^ | |
|  |  |  |  |  |  | Lower limit | Upper limit |
| 1 | Control | Psychoeducation | 7.50 | 55.02 | 1.00 | -129.23 | 144.23 |
|  |  | MBCT^b^ | -35.62 | 55.21 | 1.00 | -172.81 | 101.56 |
|  | Psychoeducation | Control | -7.50 | 55.02 | 1.00 | -144.23 | 129.23 |
|  |  | MBCT | -43.12 | 30.29 | 0.48 | -118.39 | 32.14 |
|  | MBCT | Control | 35.62 | 55.21 | 1.00 | -101.56 | 172.81 |
|  |  | Psychoeducation | 43.12 | 30.29 | 0.48 | -32.14 | 118.39 |
| 2 | Control | Psychoeducation | -1.41 | 9.53 | 1.00 | -25.11 | 22.28 |
|  |  | MBCT | -8.04 | 9.56 | 1.00 | -31.82 | 15.72 |
|  | Psychoeducation | Control | 1.41 | 9.53 | 1.00 | -22.28 | 25.11 |
|  |  | MBCT | -6.63 | 5.25 | 0.63 | -19.67 | 6.41 |
|  | MBCT | Control | 8.04 | 9.56 | 1.00 | -15.72 | 31.82 |
|  |  | Psychoeducation | 6.63 | 5.25 | 0.63 | -6.41 | 19.67 |
| 3 | Control | Psychoeducation | 1.37 | 6.41 | 1.00 | -14.56 | 17.31 |
|  |  | MBCT | -7.06 | 6.43 | 0.83 | -23.05 | 8.92 |
|  | Psychoeducation | Control | -1.37 | 6.41 | 1.00 | -17.31 | 14.56 |
|  |  | MBCT | -8.43 | 3.53 | 0.06 | -17.21 | 0.33 |
|  | MBCT | Control | 7.06 | 6.43 | 0.83 | -8.92 | 23.05 |
|  |  | Psychoeducation | 8.43 | 3.53 | 0.06 | -0.33 | 17.21 |
| Based on estimated marginal means. | | | | | | | |
| a. Adjustment for multiple comparisons: Bonferroni.  b. Mindfulnes-Based Cognitive Therapy  Table 2s. CPT-III omissions, treatment groups. | | | | | | | |

#### CPT-III Conners reacting time (correct hits)

| **Pairwise Comparisons** | | | | | | |
| --- | --- | --- | --- | --- | --- | --- |
| (I) Visit | (J) Visit | Mean difference  (I-J) | Dev,. Error | Sig^a^ | 95% confidence interval for difference^a^ | |
|  |  |  |  |  | Lower limit | Upper limit |
| 1 | 2 | 0.84 | 2.02 | 1.00 | -4.20 | 5.88 |
|  | 3 | 0.39 | 2.80 | 1.00 | -6.58 | 7.36 |
| 2 | 1 | -0.84 | 2.02 | 1.00 | -5.88 | 4.20 |
|  | 3 | -0.44 | 2.04 | 1.00 | -5.52 | 4.62 |
| 3 | 1 | -0.39 | 2.80 | 1.00 | -7.36 | 6.58 |
|  | 2 | 0.44 | 2.04 | 1.00 | -4.62 | 5.52 |
| Based on estimated marginal means. | | | | | | |
| a, Adjustment for multiple comparisons: Bonferroni.  Table 3s. CPT-III, reacting time, total group. | | | | | | |

| **Pairwise Comparisons** | | | | | | | |
| --- | --- | --- | --- | --- | --- | --- | --- |
| Visit | (I) Treatment group | (J) Treatment group | Mean difference (I-J) | Dev. Error | Sig^a^ | 95% confidence interval for difference^a^ | |
|  |  |  |  |  |  | Lower limit | Upper limit |
| 1 | Control | Psychoeducation | -5.75 | 6.40 | 1.00 | -21.67 | 10.16 |
|  |  | MBCT^b^ | -13.45 | 6.42 | 0.12 | -29.42 | 2.52 |
|  | Psychoeducation | Control | 5.75 | 6.40 | 1.00 | -10.16 | 21.67 |
|  |  | MBCT | -7.69 | 3.52 | 0.10 | -16.46 | 1.06 |
|  | MBCT | Control | 13.45 | 6.42 | 0.12 | -2.52 | 29.42 |
|  |  | Psychoeducation | 7.69 | 3.52 | 0.10 | -1.06 | 16.46 |
| 2 | Control | Psychoeducation | -5.45 | 6.96 | 1.00 | -22.76 | 11.85 |
|  |  | MBCT | -12.95 | 6.98 | 0.21 | -30.32 | 4.40 |
|  | Psychoeducation | Control | 5.45 | 6.96 | 1.00 | -11.85 | 22.76 |
|  |  | MBCT | -7.50 | 3.83 | 0.16 | -17.03 | 2.02 |
|  | MBCT | Control | 12.95 | 6.98 | 0.21 | -4.40 | 30.32 |
|  |  | Psychoeducation | 7.50. | 3.83 | 0.16 | -2.02 | 17.03 |
| 3 | Control | Psychoeducation | -9.63 | 7.55 | 0.62 | -28.41 | 9.14 |
|  |  | MBCT | -14.22 | 7.58 | 0.20 | -33.06 | 4.61 |
|  | Psychoeducation | Control | 9.63 | 7.55 | 0.62 | -9.14 | 28.41 |
|  |  | MBCT | -4.59 | 4.16 | 0.82 | -14.92 | 5.74 |
|  | MBCT | Control | 14.22 | 7.58 | 0.20 | -4.61 | 33.06 |
|  |  | Psychoeducation | 4.59 | 4.16 | 0.82 | -5.74 | 14.92 |
| Based on estimated marginal means. | | | | | | | |
| a. Adjustment for multiple comparisons: Bonferroni.  b. Mindfulnes-Based Cognitive Therapy  Table 4s. CPT-III, reacting time, treatment groups. | | | | | | | |

**Stroop, interference**

| **Pairwise Comparisons** | | | | | | |
| --- | --- | --- | --- | --- | --- | --- |
| (I) Visit | (J) Visit | Mean difference  (I-J) | Dev. Error | Sig^a^ | 95% confidence interval for difference^a^ | |
|  |  |  |  |  | Lower limit | Upper limit |
| 1 | 2 | 3.60 | 2.69 | 0.56 | -3.11 | 10.31 |
|  | 3 | 4.69 | 2.91 | 0.34 | -2.56 | 11.95 |
| 2 | 1 | -3.60 | 2.69 | 0.56 | -10.31 | 3.11 |
|  | 3 | 1.09 | 2.62 | 1.00 | -5.44 | 7.63 |
| 3 | 1 | -4.69 | 2.91 | 0.34 | -11.95 | 2.56 |
|  | 2 | -1.09 | 2.62 | 1.00 | -7.63 | 5.44 |
| Based on estimated marginal means. | | | | | | |
| a, Adjustment for multiple comparisons: Bonferroni.  Table 5s. Stroop, interference, total group. | | | | | | |

| **Pairwise Comparisons** | | | | | | | |
| --- | --- | --- | --- | --- | --- | --- | --- |
| Visit | (I) Treatment group | (J) Treatment group | Mean difference (I-J) | Dev. Error | Sig^a^ | 95% confidence interval for difference^a^ | |
|  |  |  |  |  |  | Lower limit | Upper limit |
| 1 | Control | Psychoeducation | 9.07 | 7.68 | 0.73 | -10.06 | 28.19 |
|  |  | MBCT^b^ | 2.71 | 7.65 | 1.00 | -16.36 | 21.79 |
|  | Psychoeducation | Control | -9.06 | 7.68 | 0.73 | -28.19 | 10.06 |
|  |  | MBCT | -6.35 | 3.79 | 0.30 | -15.81 | 3.10 |
|  | MBCT | Control | -2.71 | 7.65 | 1.00 | -21.79 | 16.36 |
|  |  | Psychoeducation | 6.35 | 3.79 | 0.30 | -3.10 | 15.81 |
| 2 | Control | Psychoeducation | -5.90 | 7.01 | 1.00 | -23.38 | 11.57 |
|  |  | MBCT | -7.51 | 6.99 | 0.86 | -24.94 | 9.91 |
|  | Psychoeducation | Control | 5.90 | 7.01 | 1.00 | -11.57 | 23.38 |
|  |  | MBCT | -1.61 | 3.46 | 1.00 | -10.25 | 7.03 |
|  | MBCT | Control | 7.51 | 6.99 | 0.86 | -9.91 | 24.94 |
|  |  | Psychoeducation | 1.61 | 3.46 | 1.00 | -7.03 | 10.25 |
| 3 | Control | Psychoeducation | -12.31 | 7.46 | 0.31 | -30.91 | 6.28 |
|  |  | MBCT | -16.92 | 7.44 | 0.08 | -35.47 | 1.61 |
|  | Psychoeducation | Control | 12.31 | 7.46 | 0.31 | -6.28 | 30.91 |
|  |  | MBCT | -4.61 | 3.69 | 0.65 | -13.80 | 4.58 |
|  | MBCT | Control | 16.92 | 7.44 | 0.08 | -1.61 | 35.47 |
|  |  | Psychoeducation | 4.61 | 3.69 | 0.65 | -4.58 | 13.80 |
| Based on estimated marginal means. | | | | | | | |
| a. Adjustment for multiple comparisons: Bonferroni.  b. Mindfulnes-Based Cognitive Therapy  Table 6s. Stroop, interference, treatment groups. | | | | | | | |

**TMT-B, Mistakes**

| **Pairwise Comparisons** | | | | | | |
| --- | --- | --- | --- | --- | --- | --- |
| (I) Visit | (J) Visit | Mean difference  (I-J) | Dev. Error | Sig^a^ | 95% confidence interval for difference^a^ | |
|  |  |  |  |  | Lower limit | Upper limit |
| 1 | 2 | 0.67 | 0.32 | 0.13 | -0.14 | 1.48 |
|  | 3 | 0.34 | 0.45 | 1.00 | -0.80 | 1.48 |
| 2 | 1 | -0.67 | 0.32 | 0.13 | -1.48 | 0.14 |
|  | 3 | -0.33 | 0.51 | 1.00 | -1.62 | 0.96 |
| 3 | 1 | -0.34 | 0.45 | 1.00 | -1.48 | 0.80 |
|  | 2 | 0.33 | 0.51 | 1.00 | -0.96 | 1.62 |
| Based on estimated marginal means | | | | | | |
| a, Adjustment for multiple comparisons: Bonferroni,  Table 7s. TMT-B, Mistakes, total group | | | | | | |

| **Pairwise Comparisons** | | | | | | | |
| --- | --- | --- | --- | --- | --- | --- | --- |
| Visit | (I) Treatment group | (J) Treatment group | Mean difference (I-J) | Dev. Error | Sig^a^ | 95% confidence interval for difference^a^ | |
|  |  |  |  |  |  | Lower limit | Upper limit |
| 1 | Control | Psychoeducation | 1.97 | 0.82 | 0.06 | -0.09 | 4.04 |
|  |  | MBCT^b^ | 1.75 | 0.82 | 0.12 | -0.31 | 3.81 |
|  | Psychoeducation | Control | -1.97 | 0.82 | 0.06 | -4.04 | 0.09 |
|  |  | MBCT | -0.22 | 0.35 | 1.00 | -1.11 | 0.66 |
|  | MBCT | Control | -1.75 | 0.82 | 0.12 | -3.81 | 0.31 |
|  |  | Psychoeducation | 0.22 | 0.35 | 1.00 | -0.66 | 1.11 |
| 2 | Control | Psychoeducation | 0.28 | 0.99 | 1.00 | -2.21 | 2.79 |
|  |  | MBCT | -0.55 | 0.99 | 1.00 | -3.04 | 1.94 |
|  | Psychoeducation | Control | -0.28 | 0.99 | 1.00 | -2.79 | 2.21 |
|  |  | MBCT | -0.83 | 0.43 | 0.17 | -1.91 | 0.23 |
|  | MBCT | Control | 0.55 | 0.99 | 1.00 | -1.94 | 3.04 |
|  |  | Psychoeducation | 0.83 | 0.43 | 0.17 | -0.23 | 1.91 |
| 3 | Control | Psychoeducation | -0.05 | 1.27 | 1.00 | -3.23 | 3.13 |
|  |  | MBCT | 0.30 | 1.26 | 1.00 | -2.87 | 3.47 |
|  | Psychoeducation | Control | 0.05 | 1.27 | 1.00 | -3.13 | 3.23 |
|  |  | MBCT | 0.35 | 0.54 | 1.00 | -1.02 | 1.72 |
|  | MBCT | Control | -0.30 | 1.26 | 1.00 | -3.47 | 2.87 |
|  |  | Psychoeducation | -0.35 | 0.54 | 1.00 | -1.72 | 1.02 |
| Based on estimated marginal means | | | | | | | |
| a. Adjustment for multiple comparisons: Bonferroni.  b. Mindfulnes-Based Cognitive Therapy  Table 8s. TMT-B, mistakes, treatment groups. | | | | | | | |

**Digits**

| **Pairwise Comparisons** | | | | | | |
| --- | --- | --- | --- | --- | --- | --- |
| (I) Visit | (J) Visit | Mean difference  (I-J) | Dev. Error | Sig^a^ | 95% confidence interval for difference^a^ | |
|  |  |  |  |  | Lower limit | Upper limit |
| 1 | 2 | -0.02 | 0.45 | 1.00 | -1.15 | 1.09 |
|  | 3 | -0.44 | 0.39 | 0.79 | -1.42 | 0.53 |
| 2 | 1 | 0.02 | 0.45 | 1.00 | -1.09 | 1.15 |
|  | 3 | -0.41 | 0.41 | 0.96 | -1.45 | 0.62 |
| 3 | 1 | 0.44 | 0.39 | 0.79 | -0.53 | 1.42 |
|  | 2 | 0.41 | 0.41 | 0.96 | -0.62 | 1.45 |
| Based on estimated marginal means. | | | | | | |
| a, Adjustment for multiple comparisons: Bonferroni. | | | | | | |

Table 9s, Digits, total group.

| **Pairwise Comparisons** | | | | | | | |
| --- | --- | --- | --- | --- | --- | --- | --- |
| Visit | (I) Treatment group | (J) Treatment group | Mean difference (I-J) | Dev. Error | Sig^a^ | 95% confidence interval for difference^a^ | |
|  |  |  |  |  |  | Lower limit | Upper limit |
| 1 | Control | Psychoeducation | 0.23 | 1.53 | 1.00 | -3.59 | 4.07 |
|  |  | MBCT^b^ | 0.21 | 1.53 | 1.00 | -3.61 | 4.03 |
|  | Psychoeducation | Control | -0.23 | 1.53 | 1.00 | -4.07 | 3.59 |
|  |  | MBCT | -0.02 | 0.76 | 1.00 | -1.92 | 1.86 |
|  | MBCT | Control | -0.21 | 1.53 | 1.00 | -4.03 | 3.61 |
|  |  | Psychoeducation | 0.02 | 0.76 | 1.00 | -1.86 | 1.92 |
| 2 | Control | Psychoeducation | -0.85 | 1.65 | 1.00 | -4.98 | 3.27 |
|  |  | MBCT | -0.77 | 1.65 | 1.00 | -4.89 | 3.34 |
|  | Psychoeducation | Control | 0.85 | 1.65 | 1.00 | -3.27 | 4.98 |
|  |  | MBCT | 0.08 | 0.82 | 1.00 | -1.95 | 2.12 |
|  | MBCT | Control | 0.77 | 1.65 | 1.00 | -3.34 | 4.89 |
|  |  | Psychoeducation | -0.08 | 0.82 | 1.00 | -2.12 | 1.95 |
| 3 | Control | Psychoeducation | -0.66 | 1.59 | 1.00 | -4.64 | 3.31 |
|  |  | MBCT | -1.21 | 1.59 | 1.00 | -5.18 | 2.75 |
|  | Psychoeducation | Control | 0.66 | 1.59 | 1.00 | -3.31 | 4.64 |
|  |  | MBCT | -0.54 | 0.79 | 1.00 | -2.51 | 1.42 |
|  | MBCT | Control | 1.21 | 1.59 | 1.00 | -2.75 | 5.18 |
|  |  | Psychoeducation | 0.54 | 0.79 | 1.00 | -1.42 | 2.51 |
| Based on estimated marginal means.  Table 10s. Digits, treatment groups. | | | | | | | |

a. Adjustment for multiple comparisons: Bonferroni.

b. Mindfulnes-Based Cognitive Therapy

**Letters and Numbers**

| **Pairwise Comparisons** | | | | | | |
| --- | --- | --- | --- | --- | --- | --- |
| (I) Visit | (J) Visit | Mean difference  (I-J) | Dev. Error | Sig^a^ | 95% confidence interval for difference^a^ | |
|  |  |  |  |  | Lower limit | Upper limit |
| 1 | 2 | 0.18 | 0.53 | 1.00 | -1.14 | 1.51 |
|  | 3 | -0.46 | 0.49 | 1.00 | -1.70 | 0.78 |
| 2 | 1 | -0.18 | 0.53 | 1.00 | -1.51 | 1.14 |
|  | 3 | -0.64 | 0.63 | 0.94 | -2.22 | 0.93 |
| 3 | 1 | 0.46 | 0.49 | 1.00 | -0.78 | 1.70 |
|  | 2 | 0.64 | 0.63 | 0.94 | -0.93 | 2.22 |
| Based on estimated marginal means | | | | | | |
| a, Adjustment for multiple comparisons: Bonferroni  Table 11s. Letters and Numbers, total group. | | | | | | |

| **Pairwise Comparisons** | | | | | | | |
| --- | --- | --- | --- | --- | --- | --- | --- |
| Visit | (I) Treatment group | (J) Treatment group | Mean difference (I-J) | Dev. Error | Sig^a^ | 95% confidence interval for difference^a^ | |
|  |  |  |  |  |  | Lower limit | Upper limit |
| 1 | Control | Psychoeducation | -0.09 | 1.52 | 1.00 | -3.89 | 3.70 |
|  |  | MBCT^b^ | -0.63 | 1.52 | 1.00 | -4.42 | 3.15 |
|  | Psychoeducation | Control | 0.09 | 1.52 | 1.00 | -3.70 | 3.89 |
|  |  | MBCT | -0.54 | 0.75 | 1.00 | -2.42 | 1.33 |
|  | MBCT | Control | 0.63 | 1.52 | 1.00 | -3.15 | 4.42 |
|  |  | Psychoeducation | 0.54 | 0.75 | 1.00 | -1.33 | 2.42 |
| 2 | Control | Psychoeducation | -1.23 | 1.90 | 1.00 | -5.97 | 3.49 |
|  |  | MBCT | -0.93 | 1.89 | 1.00 | -5.66 | 3.78 |
|  | Psychoeducation | Control | 1.23 | 1.90 | 1.00 | -3.49 | 5.97 |
|  |  | MBCT | 0.29 | 0.94 | 1.00 | -2.04 | 2.64 |
|  | MBCT | Control | 0.93 | 1.89 | 1.00 | -3.78 | 5.66 |
|  |  | Psychoeducation | -0.29 | 0.94 | 1.00 | -2.64 | 2.04 |
| 3 | Control | Psychoeducation | -1.14 | 1,51 | 1.00 | -4,91 | 2.63 |
|  |  | MBCT | -1.97 | 1.51 | 0.59 | -5.73 | 1.79 |
|  | Psychoeducation | Control | 1.14 | 1.51 | 1.00 | -2.63 | 4.91 |
|  |  | MBCT | -0.82 | 0.74 | 0.82 | -2.69 | 1.03 |
|  | MBCT | Control | 1.97 | 1.51 | 0.59 | -1.79 | 5.73 |
|  |  | Psychoeducation | 0.82 | 0.74 | 0.82 | -1.03 | 2.69 |
| Based on estimated marginal means | | | | | | | |
| a. Adjustment for multiple comparisons: Bonferroni.  b. Mindfulnes-Based Cognitive Therapy  Table 12s. Letters and Numbers, treatment groups. | | | | | | | |

**FEIT**

| **Pairwise Comparisons** | | | | | | |
| --- | --- | --- | --- | --- | --- | --- |
| (I) Visit | (J) Visit | Mean difference  (I-J) | Dev. Error | Sig^a^ | 95% confidence interval for difference^a^ | |
|  |  |  |  |  | Lower limit | Upper limit |
| 1 | 2 | 0.65 | 1.96 | 1.00 | -4.79 | 6.10 |
|  | 3 | -0.37 | 1.94 | 1.00 | -5.77 | 5.02 |
| 2 | 1 | -0.65 | 1.96 | 1.00 | -6.10 | 4.79 |
|  | 3 | -1.02 | 1.62 | 1.00 | -5.54 | 3.48 |
| 3 | 1 | 0.37 | 1.94 | 1.00 | -5.02 | 5.77 |
|  | 2 | 1.02 | 1.62 | 1.00 | -3.48 | 5.54 |
| Based on estimated marginal means. | | | | | | |
| a, Adjustment for multiple comparisons: Bonferroni.  Table 13s, FEIT, total group. | | | | | | |

| **Pairwise Comparisons** | | | | | | | |
| --- | --- | --- | --- | --- | --- | --- | --- |
| Visit | (I) Treatment group | (J) Treatment group | Mean difference (I-J) | Dev. Error | Sig^a^ | 95% confidence interval for difference^a^ | |
|  |  |  |  |  |  | Lower limit | Upper limit |
| 1 | Control | Psychoeducation | 1.66 | 4.53 | 1.00 | -10.93 | 14.26 |
|  |  | MBCT^b^ | 4.00 | 4.45 | 1.00 | -8.37 | 16.37 |
|  | Psychoeducation | Control | -1.66 | 4.53 | 1.00 | -14.26 | 10.93 |
|  |  | MBCT | 2.33 | 2.26 | 0.97 | -3.96 | 8.63 |
|  | MBCT | Control | -4.00 | 4.45 | 1.00 | -16.37 | 8.37 |
|  |  | Psychoeducation | -2.33 | 2.26 | 0.97 | -8.63 | 3.96 |
| 2 | Control | Psychoeducation | 7.00 | 6.52 | 0.91 | -11.13 | 25.13 |
|  |  | MBCT | 6.62 | 6.40 | 0.96 | -11.18 | 24.43 |
|  | Psychoeducation | Control | -7.00 | 6.52 | 0.91 | -25.13 | 11.13 |
|  |  | MBCT | -0.37 | 3.26 | 1.00 | -9.44 | 8.69 |
|  | MBCT | Control | -6.62 | 6.40 | 0.96 | -24.43 | 11.18 |
|  |  | Psychoeducation | 0.37 | 3.26 | 1.00 | -8.69 | 9.44 |
| 3 | Control | Psychoeducation | 2.16 | 7.21 | 1.00 | -17.87 | 22.20 |
|  |  | MBCT | 5.37 | 7.08 | 1.00 | -14.30 | 25.05 |
|  | Psychoeducation | Control | -2.16 | 7.21 | 1.00 | -22.20 | 17.87 |
|  |  | MBCT | 3.20 | 3.60 | 1.00 | -6.81 | 13.22 |
|  | MBCT | Control | -5.37 | 7.08 | 1.00 | -25.05 | 14.30 |
|  |  | Psychoeducation | -3.20 | 3.60 | 1.00 | -13.22 | 6.81 |
| Based on estimated marginal means. | | | | | | | |
| a. Adjustment for multiple comparisons: Bonferroni.  b. Mindfulnes-Based Cognitive Therapy  Table 14s, FEIT, treatment group. | | | | | | | |

**BDNF**

| **Pairwise Comparisons** | | | | | | |
| --- | --- | --- | --- | --- | --- | --- |
| (I) Visit | (J) Visit | Mean difference  (I-J) | Dev. Error | Sig^a^ | 95% confidence interval for difference^a^ | |
|  |  |  |  |  | Lower limit | Upper limit |
| 1 | 2 | 1701.36 | 2472.43 | 1.00 | -4609.42 | 8012.15 |
|  | 3 | -72.07 | 6005.07 | 1.00 | -15399.77 | 15255.62 |
| 2 | 1 | -1701.36 | 2472.43 | 1.00 | -8012.15 | 4609.42 |
|  | 3 | -1773.44 | 6326.10 | 1.00 | -17920.55 | 14373.66 |
| 3 | 1 | 72.07 | 6005.07 | 1.00 | -15255,62 | 15399.77 |
|  | 2 | 1773.44 | 6326.10 | 1.00 | -14373.667 | 17920.55 |
| Based on estimated marginal means | | | | | | |
| a, Adjustment for multiple comparisons: Bonferroni,  Table 15s, BDNF, total group. | | | | | | |

| **Pairwise Comparisons** | | | | | | | |
| --- | --- | --- | --- | --- | --- | --- | --- |
| Visit | (I) Treatment group | (J) Treatment group | Mean difference (I-J) | Dev. Error | Sig^a^ | 95% confidence interval for difference^a^ | |
|  |  |  |  |  |  | Lower limit | Upper limit |
| 1 | Control | Psychoeducation | 4286.82 | 5235.16 | 1.00 | -9075.72 | 17649.36 |
|  |  | MBCT^b^ | 3388.29 | 5223.51 | 1.00 | -9944.52 | 16721.10 |
|  | Psychoeducation | Control | -4286.82 | 5235.16 | 1.00 | -17649.36 | 9075.72 |
|  |  | MBCT | -898.53 | 1879.48 | 1.00 | -5695.83 | 3898.76 |
|  | MBCT | Control | -3388.29 | 5223.51 | 1.00 | -16721.10 | 9944.52 |
|  |  | Psychoeducation | 898.53 | 1879.48 | 1.00 | -3898.76 | 5695.83 |
| 2 | Control | Psychoeducation | 404.96 | 5625.62 | 1.00 | -13954.20 | 14764.12 |
|  |  | MBCT | 1783.33 | 5613.10 | 1.00 | -12543.88 | 16110.55 |
|  | Psychoeducation | Control | -404.96 | 5625.62 | 1.00 | -14764.12 | 13954.20 |
|  |  | MBCT | 1378.37 | 2019.66 | 1.00 | -3776.72 | 6533.47 |
|  | MBCT | Control | -1783.33 | 5613.10 | 1.00 | -16110.55 | 12543.88 |
|  |  | Psychoeducation | -1378.37 | 2019.66 | 1.00 | -6533.47 | 3776.72 |
| 3 | Control | Psychoeducation | -6445.82 | 17432.84 | 1.00 | -50942.43 | 38050.78 |
|  |  | MBCT | -49.83 | 17394.05 | 1.00 | -44447.45 | 44347.77 |
|  | Psychoeducation | Control | 6445.82 | 17432.84 | 1.00 | -38050.78 | 50942.43 |
|  |  | MBCT | 6395.98 | 6258.58 | 0.94 | -9578.78 | 22370.75 |
|  | MBCT | Control | 49.83 | 17394.05 | 1.00 | -44347.77 | 44447.45 |
|  |  | Psychoeducation | -6395.98 | 6258.58 | 0.94 | -22370.75 | 9578.78 |
| Based on estimated marginal means. | | | | | | | |
| a. Adjustment for multiple comparisons: Bonferroni.  b. Mindfulnes-Based Cognitive Therapy  Table 16s. BDNF, treatment group. | | | | | | | |
